# Supplementary material for: Improved Bioavailability of Montelukast through a Novel Oral Mucoadhesive Film in Humans and Mice
Source: Pharmaceutics. 2020 Dec 23;13(1):12. doi: 10.3390/pharmaceutics13010012 (PMC7822410; doi:10.3390/pharmaceutics13010012)
Supplement: Supplementary file 1 [file pharmaceutics-13-00012-s001.pdf]

pharmaceutics

Supplementary Materials: Improved Bioavailability of Montelukast through a Novel Oral Mucoadhesive Film in Humans and Mice

Johanna Michael, Diana Bessa de Sousa, Justin Conway, Erick Gonzales-Labrada, Rodolphe Obeid, Julia Tevini, Thomas Felder, Birgit Hutter-Paier, Horst Zerbe, Nadine Paiement and Ludwig Aigner

Table S1. Detailed clinical laboratory assessment of Phase 1 study subjects.

Test	Components	
Hematology	• Hemoglobin<br>• Platelet count<br>• WBC and differential	• Peripheral blood smear<br>• Hematocrit<br>• RBC
Serum Chemistry	• Glucose<br>• Calcium<br>• Sodium<br>• Chloride<br>• Albumin<br>• Protein<br>• Bilirubin<br>• Lactate Dehydrogenase	• AST<br>• ALT<br>• Potassium<br>• Alkaline Phosphatase<br>• Urea<br>• Uric Acid<br>• Creatinine<br>• Creatine Kinase
Urinalysis	• Bilirubin<br>• Blood<br>• Glucose<br>• pH<br>• Ketones	• Leukocytes<br>• Nitrites<br>• Protein<br>• Specific Gravity<br>• UBG
Additional Tests	Serology (HIV, Hepatitis B surface antigen, Hepatitis C antibody)	Alcohol test<br>Urine Cotinine<br>Serum hCG (females only at screening)<br>Urine hCG (females only at each period check-in)
Urine Tests for Drugs of Abuse	Marijuana, Amphetamines, Phencyclidine, Barbiturates, Cocaine, Opiates, Benzodiazepines	

Table S2. Detailed study demographics of Phase 1 study subjects.

Subject No.	Age (years)	Height		Weight		BMI	Gender	Race/Ethnicity
		(cm)	(in)	(kg)	(lb)			
1	41	172.3	67.8	84.6	186.5	28.5	Male	Black
2	45	154.8	60.9	55	121.3	23	Female	Hispanic/Latino
3	48	186.8	73.5	98.7	217.6	28.3	Male	White
4	47	173.5	68.3	74.1	163.4	24.6	Female	Black
5	46	161.5	63.6	76.8	169.3	29.4	Male	Hispanic/Latino
6	31	179.5	70.7	72.1	159	22.4	Male	Black
7	50	180.7	71.1	75.5	166.4	23.1	Male	White
8	46	182.5	71.9	96.5	212.7	29	Male	White
